# A Systematic Review of Patient-reported Outcomes in Randomized Controlled Trials of Unplanned General Surgery

**DOI:** 10.1007/s00268-015-3292-1

**Published:** 2015-11-16

**Authors:** Daniel J. Stevens, Natalie S. Blencowe, Philip J. McElnay, Rhiannon C. Macefield, Jelena Savović, Kerry N. L. Avery, Jane M. Blazeby

**Affiliations:** Centre for Surgical Research, School of Social and Community Medicine, University of Bristol, Canynge Hall, 39 Whatley Road, Clifton, Bristol, BS8 2PS UK; Division of Surgery, Head and Neck, University Hospitals Bristol NHS Foundation Trust, Bristol Royal Infirmary, Marlborough Street, Bristol, BS2 8HW UK

## Abstract

Unplanned general surgery represents a major workload and requires comprehensive evaluation with appropriate outcomes. This study aimed to summarize current reporting of patient-reported outcomes (PROs) in randomized clinical trials (RCTs) in unplanned general surgery. A systematic review identified RCTs reporting PROs in the commonest six areas of unplanned general surgery. Details of the PRO measures were examined using the CONSORT extension for PRO reporting in RCTs. Extracted information about each PRO domain included the reporting of baseline PROs, rationale for PRO selection and whether PRO findings were used in conjunction with clinical outcomes to inform treatment recommendations. The internal validity of included studies was assessed using the Cochrane risk of bias tool. 12,519 abstracts were screened and 20 RCTs containing data from 2037 patients included. Included studies used 14 separate PRO measures covering 35 different health domains. A visual analogue assessment of pain was most frequently reported (*n* = 13). Reporting of baseline PRO data was uncommon (11/35 PRO domains). The rationale for PRO data collection and a PRO-specific hypothesis were provided for 9 (25.7 %) and 5 (14.3 %) domains, respectively. Seventeen RCTs (85.0 %) used the PRO data alongside clinical outcomes to inform treatment recommendations. Of the 116 risk of bias assessments, 77 (66.0 %) were judged as high or unclear. There is a lack of well designed, and conducted RCTs in unplanned general surgery that include PROs. Future work to define relevant PROs and methods for optimal assessment are needed to inform health care decision-making.

## Introduction

Unplanned admissions to hospital with surgical problems such as appendicitis, abdominal wall hernia, and bowel obstruction represent a major volume of morbidity, healthcare expenditure and work for general surgeons [1–8]. In the UK, the National Emergency Laparotomy Audit is examining the processes and outcomes for patients undergoing emergency laparotomy. This represents important progress in improving standards of unplanned general surgical care; however, the audit and other studies have focused on clinical outcomes, and less is known about how unplanned general surgical problems impact on patient-reported outcomes. Understanding patients’ views and experiences of unplanned surgery are crucial in enabling interventions to be fully evaluated and ultimately, improving standards of care [9].

Patient-reported outcomes (PROs) may include assessment of any aspect of health, provided it comes from patients themselves. The most commonly used measures to assess PROs are health-related quality of life questionnaires, which must be valid and reliable instruments in order to provide accurate measurements [10]. The recently published PRO CONSORT extension makes recommendations to improve the way that data from these questionnaires are reported in RCTs. Improved reporting should facilitate robust interpretation of RCT results and, therefore, directly improve patient care [11]. Currently, little is known about PROs in unplanned general surgery, and whether standards of reporting are being met. It is possible that assessment of PROs in this setting is challenging because patients are often unwell, which may affect their ability to complete questionnaires before and after surgery. The aim of this study was to summarize current evidence regarding the collection of PRO data in RCTs of unplanned general surgery and to use this information to inform the design of future studies in this area.

## Materials and methods

A systematic review identified randomized controlled trials (RCTs) incorporating an assessment of PROs following unplanned surgery for conditions treated by general surgeons. Selected studies focused on RCTs because they provide high quality evidence and are expected to report outcomes of importance to patients in addition to clinical data. Hospital Episode Statistics [12] were used to identify the most common unplanned operations undertaken by general surgeons. These were appendectomy, bowel procedures (e.g. resection/repair/decompression), cholecystectomy, gastroduodenal procedures (e.g. repair of perforated peptic ulcer), drainage of perianal abscess and repair of abdominal or groyne hernia. From these, the corresponding disease areas were extrapolated: appendicitis; bowel emergencies (e.g. obstruction, inflammation or perforation); gastroduodenal emergencies (e.g. peptic ulcer); acute gallbladder disease; perianal abscess; and incarceration, obstruction or strangulation of abdominal or groyne hernia.

### Search strategy

The OVID SP version of MEDLINE, Cochrane Controlled Trials Register, Embase, PsychInfo and CINAHL databases were searched using keywords and MeSH terms relating to the anatomical location, clinical diagnosis and treatment of each of the six disease areas listed above, which were combined using the ‘OR’ operator. These were combined with standard search strategies for RCTs and PROs, using the ‘AND’ operator (Appendix 1). Searches were limited to human studies published in English between 2007 and 2012 so as to reflect current practice. Duplicate records were removed and the titles and abstracts of citations are screened for eligibility by one researcher (D.S or P.M), using pre-determined selection criteria.

### Inclusion of papers

RCTs reporting the results of PROs used to assess the diagnosis or treatment of conditions within the six disease areas described above were included. This encompassed both validated and unvalidated PRO measures (PROMs), whether as primary or secondary outcomes or part of a composite endpoint. A PRO was defined as a measure assessing physical, social or emotional aspects of health reported by the patients themselves [10]. Trials with a surgical intervention in at least one group were included, and surgical interventions were defined as “those which involve physically changing body tissues and organs through manual operation such as cutting, abrading, suturing or the use of lasers” [13]. Studies of elective surgery were excluded. Independent data extraction from full text articles meeting the inclusion criteria was performed by at least two authors (D.J.S, P.M or N.B). Where necessary, discrepancies were resolved by discussion with J.M.B.

### Data extraction

#### General study information

Details regarding the number of participants, centres and their broad geographical location were extracted. Reporting of the nature of the surgical intervention or diagnostic tool under evaluation, acquirement of ethical approval and the participant inclusion or exclusion criteria were also assessed. Each RCT was evaluated to assess whether PRO data were reported in a secondary supplementary paper to a prior main clinical trial report, or whether the PRO and clinical data were published together.

#### Patient-reported outcome assessment

The number and type of PRO measures (questionnaires) and domains (separate components of health) measured in each trial were summarized. Reporting standards were evaluated for each individual PRO domain using the PRO-specific extension to the Consolidated Standards of Reporting Trials (CONSORT) Checklist [11]. This tool provides recommendations for the reporting of PROs within RCTs, encompassing five main areas: (i) identification of PROs as primary or secondary outcomes in the abstract, (ii) provision of a PRO-specific hypothesis and relevant domains, (iii) evidence or citation of PROM instrument validity and reliability, (iv) a description of methods used to deal with missing data and (v) specification of any relevant PRO-specific limitations of study findings and generalizability of the results to clinical practice. Other CONSORT reporting standards include documentation of the primary endpoint, collection of baseline data, proportions of patients completing questionnaires at each specified time point, personnel responsible for PRO data collection, physical methods of data collection and whether PROs were reported alongside clinical outcomes.

To assess logistical aspects of PRO data collection in the unplanned setting, reporting of where and when the PRO consent was obtained from trial participants was recorded, as well as the total number of assessments performed and their time points.

#### Assessment of the risk of bias of included studies

The Cochrane risk of bias tool [14] was used to assess the methodological quality of each included RCT. This tool covers all domains of bias: random sequence generation, allocation concealment, blinding of participants and personnel, blinding of outcome assessment, incomplete data and selective reporting. A judgment of high, low or unclear risk of bias was assigned to each domain by two independent researchers (D.S., N.B. Or P.M.). Discrepancies were assessed by J.S.

### Data analysis

Results were tabulated and presented using descriptive statistics. Evidence synthesis was considered for studies using similar PROMs in the same clinical area.

## Results

**Fig. 1:**
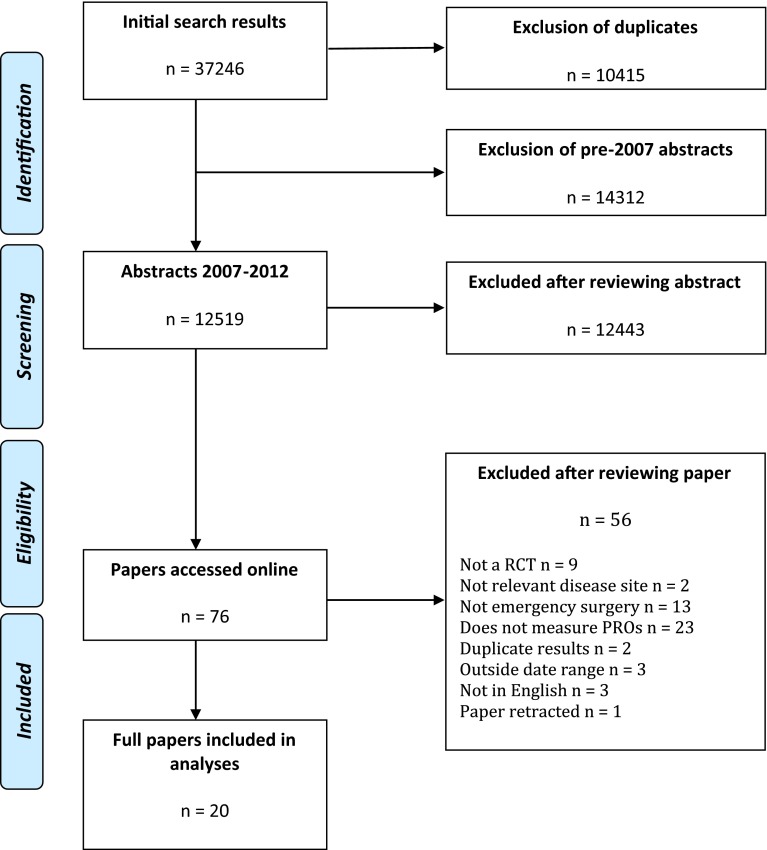
Flow diagram of papers throughout the systematic review, according to PRISMA criteria [46]

Titles and abstracts of 12,519 papers were identified, 76 full papers obtained and 20 articles included (Fig. 1) [15–34].

### Study design and participants

The 20 RCTs (of which half were single centre) included data from 2037 patients. Sixteen studies included adults (of which seven also included children), whilst three did not specify the age group of participants (Table 1). The surgical conditions under investigation were appendicitis (*n* = 13), bowel emergencies (obstruction = 2 and diverticulitis = 1, diverticulitis and/or obstruction = 1), acute gallbladder disease (*n* = 2) and peptic ulcer disease (*n* = 1). In most studies, both trial groups evaluated surgical procedures (*n* = 19), whereas one study compared surgical and conservative management strategies for appendicitis. Eighteen trials published the clinical and PRO results together in one paper. One trial published the PRO results in a separate paper to the clinical outcomes [29] and another carried out a second PRO-specific follow-up study that was published more than 10 years after the original trial [30].

**Table 1:** Details of included studies

	All studies *n* = 20
Number of participating centres	
Single	10
Multiple	6
Not specified	4
Median number of centres if multiple (range)	1 (1–25)
Geographical region of study	
Asia	7
Europe	8
Middle East	1
North America	4
Diagnoses under investigation	
Appendicitis	13
Biliary colic/acute cholecystitis	2
Large or small bowel emergencies	4
Peptic ulcer	1
Abdominal wall hernia	0
Perianal abscess	0
Types of intervention studied	
Open vs. Minimally invasive surgery	10
Surgery at different time points	4
Open vs. Endoscopy and surgery	2
Components of the same surgical procedure^a^	2
SILS^b^ vs. minimally invasive surgery	1
Surgery vs. conservative management	1
Inclusion/exclusion criteria	
Specified	19
Not specified	1
Nature of primary outcome^c^	
Mortality	0
Complications	4
Peri-operative technical outcomes	1
Treatment pathway outcomes	2
Patient-reported outcomes	2
Cost/resources	1
Not specified	10
Study participants	
Adults	9
Children	1
Both	7
Not specified	3
Mean number of participants (range)^d^	120 (37–369)
IRB or ethical approval reported	14

^a^e.g. comparing two methods of wound closure

^b^Single-Incision Laparoscopic Surgery

^c^Primary outcome fitted two categories therefore percentages calculated from denominator of 22

^d^Not reported in three of the included studies

### Patient-reported outcome assessment

A total of 35 PRO domains were measured using 14 different PROMs across the 20 RCTs, with nine RCTs including more than one PROM (Table 2). The most frequently measured PRO domain was pain, assessed using a visual analogue scale (*n* = 12). Of the studies using this measure, two provided evidence of its validity [32, 34]. Other PROMs used were the EQ-5D (*n* = 3), the Short Form-36 (*n* = 3) and the gastrointestinal quality of life index (GIQLI, *n* = 2).

**Table 2:** Details of PROMs used

	All studies *n* = 20 (%)
Number of PROMs used per study	
One	11 (55.0)
Two	6 (30.0)
Three or more	3 (15.0)
PROMs used^1^	
Gastrointestinal quality of life index (GIQLI)	2 (5.7)
Short Form-36 (SF-36)	3 (8.6)
EQ-5D	3 (8.6)
Other validated PROM	4 (11.4)
Visual analogue scale (pain)	12 (34.3)
Visual analogue scale (cosmesis)	2 (5.7)
Non-validated instrument	9 (25.7)
Consent process for PRO data collection reported	0 (0)
Location of PROM administration reported	5 (25.0)

^1^Calculated from total number of PROMs used in all 20 studies, *n* = 35

Reporting standards for each individual PRO domain measured are summarized in Table 3. Rationale for the collection of PRO data was provided for nine of 35 domains and a PRO-specific hypothesis was stated for five. Nine of the PRO domains were identified as either primary or secondary outcomes in the abstract. Evidence of instrument validity and reliability was provided for seven PROs. The personnel responsible for collecting PRO data, and methods of data collection, were reported for 15 and 19 (paper *n* = 13, telephone *n* = 1, paper and telephone *n* = 1) of the PROs, respectively. Baseline data were collected for 11 PROs but the actual number of patients completing the PROs at these assessments was never reported. The mean number of follow-up time points was 2.6 per PRO (range 1–29), and the number of patients completing PRO data at each of these follow-ups was reported 13 times from a possible 99 (13.1 %). The only study providing an explicit statement of the methods used to deal with missing data undertook statistical imputation to assess its impact [22]. Eight PROs were accompanied by a description of the potential limitations of their use and 26 interpreted the PRO data alongside clinical outcomes.

**Table 3:** Reporting standards for PRO data [11]

	Number of PROs *n* = 35 (%)
PROs identified in the abstract as a primary or secondary outcome	9 (25.7)
Rationale for PRO assessment provided	9 (25.7)
PRO hypothesis stated in background/objectives	5 (14.3)
PROs used in eligibility/stratification criteria	0 (0)
Evidence of chosen PRO instrument’s validity and reliability provided	7 (20)
Reporting of the person completing the PRO:	19 (54.3)
Method of data collection	
Paper	13 (37.1)
Telephone	1 (2.9)
Electronic	0 (0)
Other	1 (2.9)
Not reported	20 (57.1)
Explicit statement of statistical approaches for dealing with missing data	1 (2.9)
Baseline data collected	11 (31.4)
Reporting of number of patients completing PROMs at follow-up^a^	13 (13.1)
Additional analyses reported, included distinction between pre-specified and exploratory	0 (0)
PRO-specific limitations provided	8 (22.6)
PRO data interpreted alongside clinical outcomes	27 (74.3)

^a^From 99 follow-up time points

No study provided information on the consent process for completing PROMs. The location of PROM administration was reported in five studies: emergency department (*n* = 1), patient’s home (*n* = 1), postal questionnaire (*n* = 1), and on the telephone (*n* = 2).

### Risk of bias

Use of the risk of bias tool generated 116 individual assessments across the 20 trials, with 77 (66.4 %) judged to be high or unclear (Table 4).

**Table 4:** Risk of bias assessment [14]

	Issue						
	Sequence generation	Allocation concealment	Blinding (participants/personnel)	Incomplete outcome data	Blinding of outcome assessment	Selective outcome reporting	Other bias
Bertleff [19]	Low risk	Unclear risk	High risk	Low risk	High risk	High risk	
Blakely [25]	Low risk	High risk	High risk	Low risk	High risk	Low risk	
Cheung [20]	Low risk	Unclear risk	High risk	High risk	High risk^a^	High risk	
Clarke [18]	Unclear risk	Unclear risk	Low risk	High risk	Low risk	High risk	
Goudar [26]	Unclear risk	Unclear risk	High risk	High risk	Unclear risk	Low risk	
Hansson [21]	High risk	High risk	High risk	Low risk	Unclear risk	Low risk	
Kaplan [34]	High risk	High risk	Unclear risk	Low risk	Low risk	Low risk	
Kargar [28]	Low risk	Unclear risk	High risk	Unclear risk	High risk	High risk	
Klarenbeek [33]	Low risk	Low risk	Low risk	Low risk	Low risk	Low risk	
Kouhia [24]	Unclear risk	High risk	Unclear risk	Low risk	Unclear risk	Unclear risk	
Macafee [22]	Low risk	Low risk	High risk	Low risk	High risk	Low risk	Low risk
Malik [15]	Unclear risk	High risk	Unclear risk	Unclear risk	Unclear risk	Unclear risk	
Alam Jan [27]	Unclear risk	High risk	High risk	High risk	High risk	Low risk	
Ricca [16]	Unclear risk	Unclear risk	Unclear risk	Unclear risk	Low risk	Unclear risk	Low risk
Schurman [29]*	*	*	*	*	*	*	*
Suresh [31]	Low risk	High risk	Unclear risk	Unclear risk	Unclear risk	Unclear risk	
van der Wal [30]	Low risk	Unclear risk	Unclear risk	Unclear risk	Unclear risk	High risk	
van Hooft [17]	Low risk	Low risk	High risk	Low risk	Unclear risk	Low risk	
Yadav [23]	Unclear risk	High risk	Unclear risk	Unclear risk	Unclear risk	Unclear risk	
Yuen Bun Teoh [32]	Low risk	Unclear risk	Unclear risk	Unclear risk	Low risk	Low risk	

* Pilot RCT—not appropriate to assess RoB

^a^Low risk for mortality and high risk for all other outcomes

### Data analysis

Data from the PROs were not synthesized because of the heterogeneity of PROMs, conditions and interventions.

## Discussion

This systematic review of PRO reporting in RCTs in unplanned general surgery identified 20 eligible trials. None of the measures used to assess PROs were specific for unplanned surgical settings. Just 11 studies reported baseline data and the proportion of patients completing follow-up assessments was rarely documented. Overall, the methodological quality of the included RCTs was judged to be poor and reporting of PRO data did not conform to CONSORT standards. The lack of high quality data means that more research to evaluate PROs in this setting is needed.

The importance of incorporating patients’ views about outcome measurement and reporting within RCTs has been highlighted by recent guidance [9, 35, 36]. PROs are useful because they avoid the inherent bias that may occur when assessments are performed by observers. In addition, they may detect issues of importance to patients that may be overlooked in routine clinical follow-up. This review identified a total of 14 unique PROMs which were used 35 times. Few studies used the same measure at similar time points, making it impossible to synthesize outcomes. Others have highlighted these issues and the difficulties of combining PROMs [37]. One potential solution to the problem of heterogeneity of outcomes is to develop and use a core outcome set. A core outcome set is a minimum set of agreed outcomes to be measured and reported in all trials of a particular treatment or condition [37]. The Core Outcome Measures in Effectiveness Trials (COMET) initiative emphasizes this approach as a way of aiding data synthesis and reducing reporting bias [38]. Core outcome sets aim to include outcomes of importance to all stakeholders, including patients [39]. Core outcome sets are being developed in various surgical contexts including esophageal cancer, breast reconstruction, colorectal and obesity surgery [40]. A core outcome set for unplanned general surgery may be helpful in addressing the issues outlined above and improve evidence synthesis across trials. Methods for including PROs in core outcome sets have been previously established [37].

Limitations of this review include the application of several restrictions to the search criteria. First, databases were only searched between 2007 and 2012. Whilst it is possible that the methodological quality of trials and standards of PRO reporting differed in previous years, evidence supports a general trend of improved reporting over time [41]. Second, searches were limited to six broad, but discrete disease sites. This was done because unplanned surgery publications are not consistently indexed in literature databases, and there are no validated search strategies specifically developed for this area. Hospital Episode Statistics data was therefore used to identify the six most common diseases presenting as unplanned admissions to surgical services. It is possible that RCTs involving less common conditions were inadvertently missed. Third, included study designs were limited to RCTs only. This was necessary in order to ensure the review was manageable; 12,519 abstracts were identified and this number would have been larger if a specific RCTs search strategy had not been applied. Another reason for including only RCTs was that the review aimed to assess the quality of reporting of PROs in unplanned surgery. To the authors’ knowledge, the PRO extension to the CONSORT checklist is currently the only available tool for assessing reporting standards—and this is designed specifically for RCTs. The final limitation is that no PROMs specific for unplanned general surgery were identified, meaning that content validity for patients with these conditions could not be established. Further work may need to ascertain whether existing PROMs are of relevance and importance to such patients, and explore whether a PRO-specific tool for unplanned non-trauma general surgery is required.

Patient-reported outcomes have also been evaluated in other unplanned settings such as intensive care units, traumatic brain injury, acute medical admissions, wartime injuries, and inpatient rapid response teams [42–45]. Many similar problems were identified including a lack of PROMs specific to unplanned conditions, failure to collect baseline data and heterogeneity in follow-up time points. The relative dearth of high quality studies assessing PROs in all these acute settings may reflect logistical difficulties and the resource intensive nature of collecting such data in this environment. These problems may help to explain the poor reporting standards amongst published RCTs, which makes meaningful interpretation of PRO data difficult. The paucity of high quality RCTs identified in this review make it difficult to reliably use PRO data when evaluating the interventions in these studies, meaning more research is needed.

## Appendix 1

### Search strategy

#### Patient-reported outcomes

1. “Quality of Life”/.
2. Quality of life.tw.
3. Qol.tw.
4. Hrql.tw.
5. Hrqol.tw.
6. “Outcome Assessment (Health Care)”/
7. Patient-reported outcome.tw.
8. Patient-reported outcome.tw.
9. Patient-reported outcome measure.tw.
10. Patient-reported outcome measure.tw.
11. Health Status/
12. Health status.tw.
13. PRO
14. PROM
15. Pain/
16. Physical function.tw.
17. Fatigue/
18. Well-being.tw.
19. Well-being.tw.
20. Health surveys/
21. Treatment outcome/
22. Euroqol.tw.
23. EQ-5D.tw.
24. EQ-3D.tw.
25. Gastrointestinal quality of life index.tw.
26. GIQLI.tw.
27. SF-36.tw.
28. Or/1-27

#### Randomized trials

1. Randomized Controlled Trials as Topic/
2. Randomized controlled trial/
3. Random Allocation/
4. Double-Blind Method/
5. Single-Blind Method/
6. Clinical trial/
7. Clinical trial, phase i.pt
8. Clinical trial, phase ii.pt
9. Clinical trial, phase iii.pt
10. Clinical trial, phase iv.pt
11. Controlled clinical trial.pt
12. Randomized controlled trial.pt
13. Multicentre study.pt
14. Clinical trial.pt
15. Exp Clinical Trials as topic/
16. Or/1-15
17. (clinical adj trial$)
18. ((singl$ or doubl$ or treb$ or tripl$) adj (blind$3 or mask$3))
19. PLACEBOS/
20. Placebo$.tw
21. Randomly allocated.tw
22. (allocated adj2 random$).tw
23. Or/17-22
24. 16 or 23
25. Case report.tw
26. Letter/
27. Historical article/
28. Or/25-27
29. 24 not 28

#### Abscess

1. Anal.tw
2. Anus.tw
3. In-ano.tw
4. Perianal.tw
5. Or/1-4
6. Abscess/
7. Fistul$
8. Drain$.tw
9. Pus.tw
10. Suppuration/
11. Or/6-10
12. 5 and 11

#### Appendicitis

1. Appendix/
2. Appendix.tw
3. Appendectomy/
4. Appendicectomy.tw
5. Appendicitis/
6. Appendicitis.tw
7. Or/1-6
8. 7 NEAR/10 perforat*
9. 7 NEAR/10 ruptured
10. Or/1-9

#### Gallbladder

1. Cholecystolithiasis/
2. Cholecystitis/
3. Cholelithiasis/
4. Pancreatitis/
5. Pancreatitis.tw
6. Biliary colic.tw
7. Empyema.tw
8. Cholecystectomy/
9. Cholecystostomy/
10. Choledochostomy/
11. Or/1-10

#### Gastric/duodenal

1. 1.Peptic Ulcer/
2. 2.Gastrointestinal H*morrhage/
3. 3.Peptic Ulcer Perforation/
4. 4.Stomach Ulcer/
5. 5.Peptic.tw
6. Gastric.tw
7. Duodenal.tw
8. Stomach.tw
9. Or/5-8
10. Ulcer.tw
11. 9 and 10
12. Or/1-4 or 11
13. 12 NEAR/10 perforation
14. 12 NEAR/10 rupture
15. 12 NEAR/10 haemorrhage
16. 12 NEAR/10 haemorrhage
17. 12 NEAR/10 bleeding
18. Or/13-17 or 12

#### Hernia

1. Hernia, Abdominal/
2. Hernia, Obturator/
3. Inguinal.tw
4. Femoral.tw
5. Ventral.tw
6. Obturator.tw
7. Umbilical.tw
8. Or/3-7
9. Herni$.tw
10. 8 and 9
11. Or/1-2 or 10
12. 11 NEAR/10 perforation
13. 11 NEAR/10 rupture
14. 11 NEAR/10 obstruction
15. Or/12-14 or 11
16. Herniorrhaphy/
17. Herniorrhaphy.tw
18. Hernia surgery.tw
19. (laparoscop$ adj25 herni$).tw
20. (open adj10 herni$).tw
21. (darn adj10 herni$).tw
22. (mesh adj10 hern$).tw
23. (traditional adj10 herni$).tw
24. (plug adj10 herni$).tw
25. (lichtenstein adj10 herni$).tw
26. Or/16-25
27. 15 and 26

#### Large/small bowel

1. Divertic$.tw
2. Diverticulum, Colon/
3. Intestinal Obstruction/
4. Intestinal Perforation/
5. Colonic Diseases/
6. Cecal Diseases/
7. Tissue adhesions/
8. Or/1-7
9. 8 NEAR/10 obstruction.tw
10. 8 NEAR/10 perforation.tw
11. 8 NEAR/10 peritonitis.tw
12. 8 NEAR/10 bleeding.tw
13. 8 NEAR/10 haemorrhage.tw
14. 8 NEAR/10 haemorrhage.tw
15. Hartman$.tw
16. Surgical Procedures, Operative/
17. Laparoscopy/
18. Resect$.tw.
19. Operat$.tw.
20. Surg$.tw.
21. Laparo$.tw
22. Or/9-21
23. 8 and 22
